# Intraperitoneal migration of an intrauterine device (IUD): A case report

**DOI:** 10.1016/j.amsu.2021.102547

**Published:** 2021-07-08

**Authors:** Hicham Benaguida, Hamza Kiram, Ely Cheikh Telmoudi, Btissam Ouafidi, Mustapha Benhessou, Mohamed Ennachit, Mohamed Elkarroumi

**Affiliations:** a Resident Doctor in Obstetrics and Gynecology Department, Univesity Hospital Center Ibn Rochd, Casablanca, 20100, Morocco; b Professor in Obstetrics and Gynecology Department, Univesity Hospital Center Ibn Rochd, Casablanca, 20100, Morocco; c Head of Service in Obstetrics and Gynecology Department, Univesity Hospital Center Ibn Rochd, Casablanca, 20100, Morocco

**Keywords:** Intrauterine device, Migration, Intraperitoneal, Laparoscopy

## Abstract

**Introduction:**

The IUD is one of the most widely used reversible, long-term contraceptive methods in the world. About 80% of IUDs are found in the peritoneal cavity after uterine perforation.

**Case report:**

A 27-year-old female patient presented with chronic pelvic pain with minimal metrorrhagia for 8 months on IUD. On examination, there was no IUD thread. Pelvic ultrasound showed a hypoechoic, heterogeneous, poorly limited formation measuring 3 × 2.68 cm. Abdominal-pelvic CT scan showed hyperdense supravesical material surrounded by a hypo-dense, well-limited collection measuring 26 × 25 mm. Laparoscopy showed an anterior peritoneal collection above the bladder containing the IUD, a uterus, adnexa, and a bladder without abnormality. The IUD was removed after incision of the collection and aspiration of the pus.

**Discussion:**

The IUD is one of the most widely used long-term reversible contraceptive methods in the world. But like any foreign body, it can present complications, notably migration after uterine perforation, which remains rare, and even rarer peritoneal localization. The clinical diagnosis is not always obvious, and additional examinations are necessary to locate the device, including endovaginal ultrasound, a CT scan or magnetic resonance imaging.

WHO recommends surgical removal of the migrated IUD by minimally invasive methods, including hysteroscopy, cystoscopy, colonoscopy, or laparoscopy, depending on the location of the IUD.

**Conclusion:**

IUDs are effective contraceptive measures, and the majority of patients with uterine perforation by IUD migration are asymptomatic. Diagnosis is based on a thorough gynecologic analysis and appropriate radiologic imaging.

## Highlights

• The IUD is one of the most widely used long-term reversible contraceptive methods in the world.
• Perforation with migration is rare but is one of the most serious complications.
• Clinical diagnosis is not always evident and needs additional examinations to locate the device (Ultrasound, CT scan, MRI).
• Who recommends surgical removal of migrated DIU as soon as possible, using minimally invasive methods, including hysteroscopy, laparoscopy, depending on the location of the DIU.

## 1 Introduction

Intrauterine devices are used by about 14% of women worldwide and up to 27% in some regions of the world [1]. Copper- or levonorgestrel-based IUDs are among the most effective methods of contraception, with failure rates of less than 1% during the first 12 months of use [2]. Evidence-based information about the benefits and harms of the IUD is needed to inform decision making and dispel myths and misconceptions. Available evidence suggests that pregnancy rates, adverse events, and discontinuation because of side effects during the first two years of prolonged IUD use are low and may not be clinically significant. However, insertion can lead to some complications, mainly when the rules of use are not followed, such as infection, expulsion, or perforation [3].

Perforation is exceptional but one of the most serious complications. Indeed, after a perforation, the IUD can be localized in various neighboring organs. Ectopic localization in the pouch of Douglas, omentum, mesentery, colon and bladder have been described [4,5].

We report a new case of intraperitoneal IUD migration, diagnosed 1.5 years after insertion. Laparoscopic surgery was performed to remove the IUD, which was embedded in the peritoneum pre- and supravesical. This work has been reported with respect to the SCARE 2020 criteria [6].

## 2 Case report

**Fig. 1:**
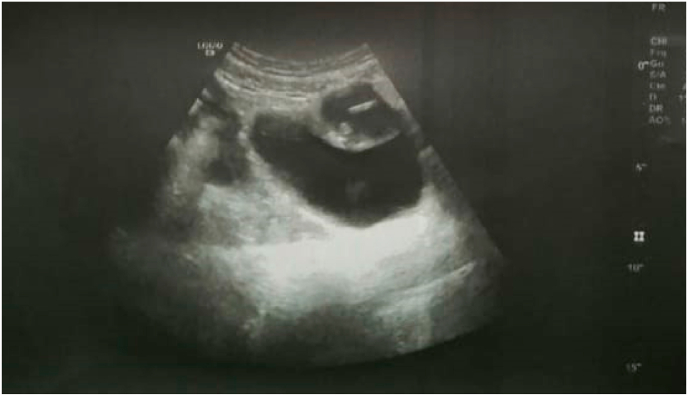
Suprapubic pelvic ultrasound: Appearance suggestive of a pre-vesical migration of an IUD with a collection around it.

Patient aged 27 years, mother of a child with no particular pathological history. She had had an IUD inserted a year and a half before her consultation with the aim of contraception. The patient reported chronic pelvic pain in the hypogastrium and several episodes of minimal metrorrhagia after 8 months of IUD insertion without fever or associated urinary or digestive signs, and wished to switch to the oral contraceptive pill. An attempt to remove the IUD during the consultation was unsuccessful because of the absence of an IUD thread. Pelvic ultrasound showed a hypoechoic, heterogeneous, ill-defined formation with irregular contours centered by a bilobed hyperechoic structure with a shadow cone measuring 3 × 2.68 cm (Fig. 1).

**Fig. 2:**
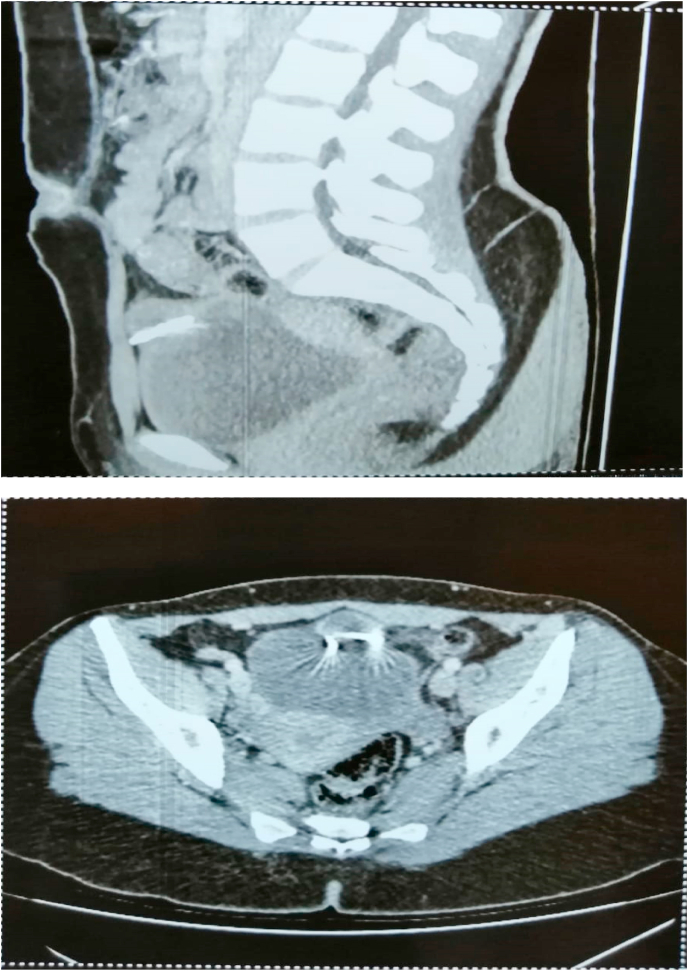
Abdomino-pelvic CT showed on a sagittal section and an axial section: an anteverted uterus with an IUD above the bladder associated with a collection came into contact with the anterior wall of the bladder with conservation of the separation line.

An abdominal-pelvic CT scan showed hyperdense, artifact-generating supravesical material surrounded by a round, well-limited hypodense collection, peripherally enhanced after PDC injection, measuring 26 × 25 mm. This collection was in contact with the anterior wall of the bladder with preservation of the separation line (Fig. 2).

**Fig. 3:**
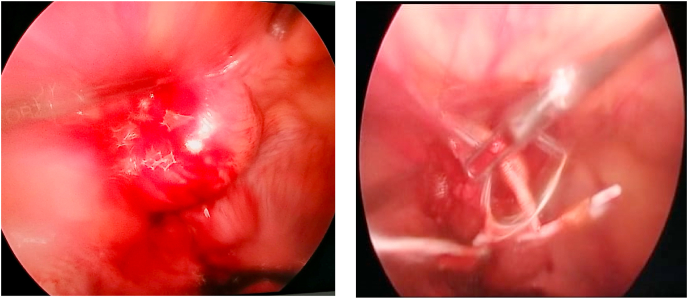
Exploration by laparoscopy.(a). A supra-vesical peritoneal collection, (b). Extraction of an IUD migrated intraperitoneally after incision of the collection.

Laparoscopy showed an anterior peritoneal collection above the bladder with intra-abdominal migration of the IUD, a uterus of normal size and appearance with no evidence of perforation, and the adnexa and bladder were free of any lesions (Fig. 3). The IUD was removed after incision of the collection and aspiration of the pus; the material was sent for bacteriological study at the end of the operation.

The patient was followed in our department for one year after removal of the IUD with good clinical improvement.

## 3 Discussion

The intrauterine device (IUD) is one of the most widely used methods of long-term reversible contraception in the world [1,7]. Bioactive, copper, copper-silver, or progestin IUDs are the most commonly used because of their better tolerability, but like any foreign body, the IUD can present risks and complications. These include not only abdominal or pelvic pain, abnormal bleeding, dysmenorrhea, unplanned pregnancy, and spontaneous abortion, especially in the first few months after insertion, but also expulsion, the incidence of which can be as high as 25%, infection, and migration after uterine perforation, which remains rare [8].

About 80% of IUDs are found in the peritoneal cavity after perforation. Migration into the surrounding organs is a rare but serious complication after perforation [9]. This migration can take several directions; it usually occurs in the peritoneal cavity and rarely in the surrounding pelvic organs, mainly the bladder, rectosigmoid, omentum, peritoneum, bladder, appendix, small intestine, adnexa, and iliac vein [10]. Perforation can be primary and occur during insertion, which is usually associated with severe abdominal pain that should attract the attention of the physician [11]. While secondary perforation is a late event, supposedly due to progressive pressure and necrosis of the uterine wall [12].

Symptomatology varies depending on the site of migration and the type of IUD. In our case, the patient presented with chronic pelvic pain for 8 months with several episodes of minimal metrorrhagia. In the literature, 85% of reported cases of perforation were asymptomatic at the time of diagnosis [13]. However, in some cases, the diagnosis may be made by the appearance of clinical signs such as fever, abdominal pain, diarrhea or urinary tract infection, or even serious complications such as occlusive syndrome or peritonitis due to perforation of a hollow organ.

The clinical diagnosis is not always obvious, and further investigations are necessary to locate the device. Imaging has a great advantage in the topographic diagnosis of a migrated IUD. Abdominopelvic ultrasound is then indicated as the first choice [14]. It shows an empty uterine cavity or a parauterine IUD. Sometimes it does not find an IUD but cannot confirm a uterine perforation. Endovaginal ultrasound is a better way to assess uterine emptiness. If the IUD is not seen by ultrasound, refer to recommendations that prescribe a computed tomography (CT) or magnetic resonance imaging (MRI) scan. Abdominal radiography without preparation has lost its place in this indication in the face of these new imaging techniques [15,16].

The World Health Organization recommends surgical removal of the migrated IUD as soon as possible, even in asymptomatic patients. The recommendation is to use minimally invasive methods if possible, including hysteroscopy, cystoscopy, colonoscopy, or laparoscopy, depending on the location of the IUD. If it is embedded in an organ such as the bladder or bowel, invasive removal is not recommended, but rather an exploratory laparotomy. Similarly, if the device is buried near a blood vessel or is not fully visualized, more invasive methods are recommended by an experienced surgeon [[17], [18], [19]].

## 4 Conclusion

IUDs are effective contraceptive measures, and many patients with uterine perforation by IUD migration may present with symptoms, but up to 85% are asymptomatic. The diagnosis of a migrated IUD should be based on a thorough gynecologic analysis and appropriate radiologic imaging. Prospective investigation of displaced and migrated IUDs is needed, especially for women with a history of scarred uterus following cesarean section or myomectomy. Surgical removal is a first-line option to avoid serious complications; hysteroscopy or laparoscopy remains appropriate.

## Ethical approval

I declare on my honor that the ethical approval has deen exempted by my establishment.

## Author contribution

Hicham Benaguida: writing the paper, Hamza Kiram: study concept, Ely Cheikh Telmoudi: Corresponding author writing the paper and operating surgeon, Btissam Ouafidi: study concept, Mustapha Benhessou: study concept, Mohamed Ennachit: study concept, Mohamed Elkarroumi: correction of the paper and operating surgeon.

## Registration of research studies

Researchregistry2464.

## Guarantor

DR TELMOUDI ELY CHEIKH.

## Consent

Written informed consent was obtained from the patient for publication of this case report and accompanying images. A copy of the written consent is available for review by the Editor-in-Chief of this journal on request.

## Provenance and peer review

Not commissioned, externally peer-reviewed.

## Funding

None.

## Declaration of competing interest

The authors declare that they have no known competing financial interests or personal relationships that could have appeared to influence the work reported in this paper.
